# Transitional Electrodes in Electrowetting-Based Droplet Dispensing

**DOI:** 10.3390/bios14010044

**Published:** 2024-01-14

**Authors:** Wei Wang, Qijun Cai, Shangzhe Xu, Xucan Chen

**Affiliations:** 1 MOE Key Laboratory of Material Physics and Chemistry under Extraordinary Conditions, School of Physical Science and Technology, Northwestern Polytechnical University, Xi’an 710129, China; 2 Computer Science Department, Georgia State University, Atlanta, GA 30301, USA

**Keywords:** digital microfluidics, electrowetting-on-dielectric, droplet dispensing, volumetric consistency, transitional electrodes

## Abstract

Digital microfluidic systems based on electrowetting-on-dielectric technology, particularly valuable in producing and manipulating microdroplets steadily and consistently, have experienced notable advancements in recent years. In this paper, experimental characterizations reveal that simply adding one transitional electrode between the reservoir and the splitting electrode improves the volumetric consistency and reproducibility for droplet dispensing. The volumetric coefficient variation of the consecutively dispensed droplets from a non-refilling reservoir decreases by 1% after the addition of one transitional electrode, with no extra external apparatus. This work provides a straightforward yet effective approach to the improvement of digital microfluidic systems and micro total analysis systems.

## 1. Introduction

The miniaturization of micro-electro-mechanical systems in the microelectronics industry has revealed its significance in the integration and automation of many processes into one single device or system. This gives rise to tremendous performance improvements in mechanical engineering and fluid mechanics with higher precision, throughput, and functionality. Among these advancements, the origin of microfluidic technology can be traced back to the silicon-based gas chromatograph developed by Stanford University in the 1970s [1] and the inkjet printer developed by IBM Corporation [2,3]. The formal conceptualization of microfluidics emerged in the early 1990s [4]. Over the past decades, the connotation of microfluidics has gradually enriched and finally formed a research direction covering many cutting-edge fields involving biochemical analysis [5,6,7], point-of-care detection [8], clinical analysis [9], molecular diagnosis [10], and single-cell analysis [11]. In the field of microfluidic technology, researching the method of producing and controlling the microdroplets is more than significant.

Digital microfluidics based on electro-wetting-on-dielectric (EWOD) presents a promising approach for manipulating reagent-contained droplets across a pre-designed routine, as EWOD technology greatly improves the manipulation accuracy and selectivity of droplets. Therefore, the volumetric consistency of the droplets produced within the EWOD system largely determines the accuracy of the final detection results obtained in micro-total analysis systems (μ-TAS). For example, in a lab-on-a-chip system that detects the concentration of glucose in humans, the volume of microdroplets generated by the system cannot fluctuate by more than ±2% [12]. Only EWOD devices that can accurately and reproducibly generate simple droplets of the required volume can meet the precision requirements in practical applications and perform the advantages of digital microfluidic systems. However, affected by the Rayleigh–Plateau instability and the resulting oscillation on the liquid–air interface, the continuous alteration of liquid morphology during droplet generation (pulling out first, shrinking in the middle to form a thin neck then, and finally pinching off to generate droplets) renders the process highly susceptible to external disturbances [13,14,15,16]. Consequently, a minute alteration in initial conditions or the introduction of stochastic disturbances can exert a significant impact on the volumetric variation of consecutively dispensed droplets for traditional EWOD devices.

Early researchers chose to abandon the droplet generation function of the EWOD device and use an off-chip micropump with higher precision to inject liquid into the digital microfluidic device instead. Ren et al. [17] and Gong et al. [18] further set up a capacitive sensing component in the liquid injection region. During the injection of liquids into the EWOD device by the micropump or the on-chip dispensing process, the capacitance detection component monitors the volume of liquid that has entered the device in real-time, and feeds the result back to the control system to dynamically adjust the liquid injection rate or the voltage applied to EWOD electrodes. With this setup, the volumetric fluctuation of the microdroplets generated within the EWOD device could be reduced to about ±2%. However, the introduction of new modules, like the external micropump, liquid detection components, or feedback loops, greatly increases the system complexity, reduces the integration level, and increases the production costs in the meanwhile, which is contrary to the design purposes and technical advantages of the digital microfluidic system. In addition, through computer simulation, Berthier et al. found that the volumetric accuracy of dispensed droplets can be improved by optimizing parameters such as electrode size, gap height between two plates, and driving voltage of the EWOD device [16]. However, this method is not universal for different research conditions, since parameters like height and voltage are prefixed and inalterable, it cannot provide general recommendations for improvement applicable to various EWOD devices, which could be extremely work-intensive for digital microfluidic systems involving the variation of various parameters. An alternative function, called passive dispensing, was described by Wheeler et al. [19]. Hydrophilic sites were built on a hydrophobic surface to help the formation of sub-droplets, but difficulties can be anticipated for multi-step processes since the droplet becomes stuck after dispensing. Moreover, substrates with chemical patterns on the surface were also studied for the modulation of droplet morphology [20,21,22]. The shape of splitting electrode in the EWOD device was also meticulously designed (e.g., into dumbbell shape) to increase the controllability and stability of dispensing process [23,24]. However, this method requires an introduction of sub-electrodes with fine structures that put extra demand for the manufacturing process.

In this paper, experimental characterizations reveal the efficiency of a straightforward modification in electrode design, aimed at enhancing the stability and reproducibility of the droplet dispensing process with no peripherals other than electrical connections. By simply adding one transitional electrode between the reservoir and the splitting electrode, the volumetric coefficient variation of the consecutively dispensed droplets from a non-refilling reservoir can be greatly decreased. This observation confirms a simple and reliable approach to improving the volumetric consistency and accuracy of digital microfluidic systems. Moreover, additional experiments indicate that different parameters of the transitional electrode, like aspect ratio and gap height, have a great influence on the coefficient of variation over the volume of consecutively dispensed droplets as well, which reveals the potential industrial application value of precision instrument manufacturing.

## 2. Materials and Methods

**Figure 1:**
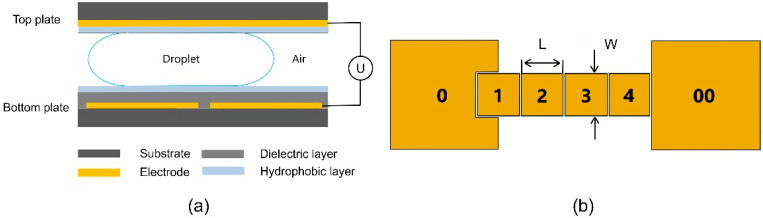
Schematics of the EWOD device used in this work. (**a**) Side view. (**b**) Top view of actuation electrodes in the bottom plate.

This work uses a two-plate EWOD structure with square-shaped electrodes, as depicted in Figure 1a. The glass coated with a 120-nm-thick indium tin oxide (ITO) conductive film was ultrasonically cleaned in acetone, isopropanol, and deionized water in sequence for 10 min each. The cleaned ITO glass was then blown dry by clean nitrogen gas and heated on a hot plate surface at 120 °C for 30 min. The ITO coating on bottom substrates was then patterned with lithography and wet etching into designed two-dimensional electrode arrays as shown in Figure 1b. In this experiment, 1.5 μm SU8-2002 (MicroChem, Round Rock, TX, USA) and 60 nm Teflon AF-1600 (DuPont, 400S2-100-1, Wilmington, DE, USA) were spin-coated successively onto the lithographically patterned bottom substrate to prepare the insulating dielectric layer and the hydrophobic layer in the EWOD structure, respectively. Another piece of ITO glass was taken as the top plate after cleaning, drying, and spin-coating the Teflon layer. Double-sided tapes were pasted on the edge of the top plate to align the top and bottom plates, defining the height of the gap between the parallel plates. After adding liquid above the electrode of the bottom plate with a micropipette, the two plates and press the top plate were aligned lightly to ensure a firm bonding and a uniform gap height. After bonding steps, the EWOD electrodes in the bottom plate were connected to an external supply through a laboratory-made relay while the ITO layer in the top plate was kept grounded.

In this work, unless otherwise stated, the driving voltage is a sinusoidal alternating current signal with a frequency of 1 kHz and an amplitude of 60 V_rms_. The electrode sequence on the bottom plate is illustrated and numbered in Figure 1b. The size of electrode 0 and electrode 00, as liquid reservoirs, is 8 mm × 8 mm while electrodes 1–4 are 2 mm × 2 mm. The gap between adjacent electrodes is 50 μm. The dispensing process of deionized water droplets was captured by a microscope (TE2000-U, Nikon, Japan) with the top-view area of droplets recorded using ImageJ2 software (National Institutes of Health, Bethesda, MD, USA). Considering that the rim width of the droplet’s top view is much smaller than its radius due to a small gap height, the surface curve caused by the Young–Laplace effect is negligible and the volume of the droplets can be approximately calculated as the product of the area of the droplet and the gap height. To express the volumetric consistency of droplets more intuitively and comparatively, the coefficient of variation (*CV*) over the volume of consecutively dispensed droplets is used here to characterize the volumetric stability of droplets generated by different devices. In this regard, the coefficient of variation was calculated by dividing the standard deviation with the mean volume *μ* of consecutively dispensed droplets, yielding

$$CV=\frac{1}{\mu }\sqrt{\frac{1}{N}\overset{N}{\underset{i=1}{\sum }}{\left(x_{i}-\mu \right)}^{2}}=\sqrt{\frac{1}{N}\overset{N}{\underset{i=1}{\sum }}{\left(\frac{x_{i}-\mu }{\mu }\right)}^{2}}$$

where $x_{i}$ is the volume of the *i*-th droplet in total *N* droplets. Ten droplets were dispensed consecutively from a non-replenishing reservoir in each measurement. To decrease the accidental error, each data proposed in this work was averaged over ten individual measurements. Consequently, in this work, a smaller *CV* indicates higher volumetric consistency and stability of the corresponding droplet collection and dispensing process.

## 3. Results

**Figure 2:**
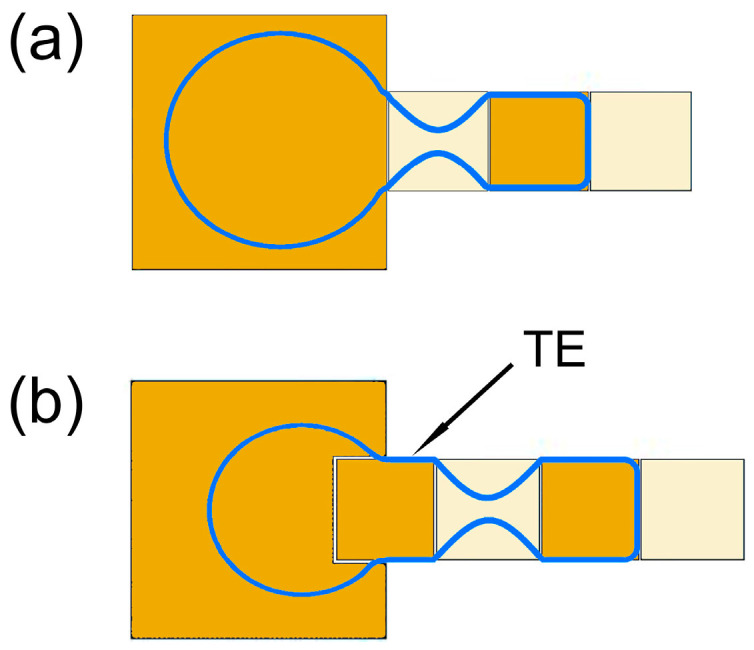
Schematics of the droplet dispensing processes (**a**) without and (**b**) with a transitional electrode (TE). The blue contour demonstrates the shape of the liquid. The dark golden electrodes are applied to the actuation voltage while the light ones are grounded.

In the EWOD device, the droplet generation process includes three processes: liquid pulling from the reservoir to spread along the driving electrodes, necking, and breaking to form a separate droplet. For EWOD electrode arrays without the transitional electrode, the dispensing process is featured in Figure 2a. Briefly, electrode 00 was used as the reservoir electrode. One large water droplet, which is called the mother droplet, is initially located above the reservoir electrode 00 and partially overlaps the driving electrode 4. When driving electrode 4 and driving electrode 3 are connected to an actuation voltage, a portion of the mother droplet is pulled over the actuated electrodes. This process is called liquid pulling. Then, keep the driving electrode 3 powered on, connect the reservoir electrode 00 to the driving voltage, and ground the driving electrode 4. Then, the liquid above the driving electrode 4 gradually shrinks from the middle to form a thin neck under the action of surface tension and electrocapillary action. This is the necking process of the liquid. Finally, the thin neck is pinched off due to the Rayleigh-Plateau instability, and the liquid above the driving electrode 4 is merged back into the mother droplet in the reservoir under the combined action of surface tension and electrocapillary force, respectively, forming a droplet above the driving electrode 3. Hereinafter, the driving electrode corresponding to the occurrence position of the liquid breaking process is referred to as the splitting electrode, matching electrode 4 in Figure 2a.

**Figure 3:**
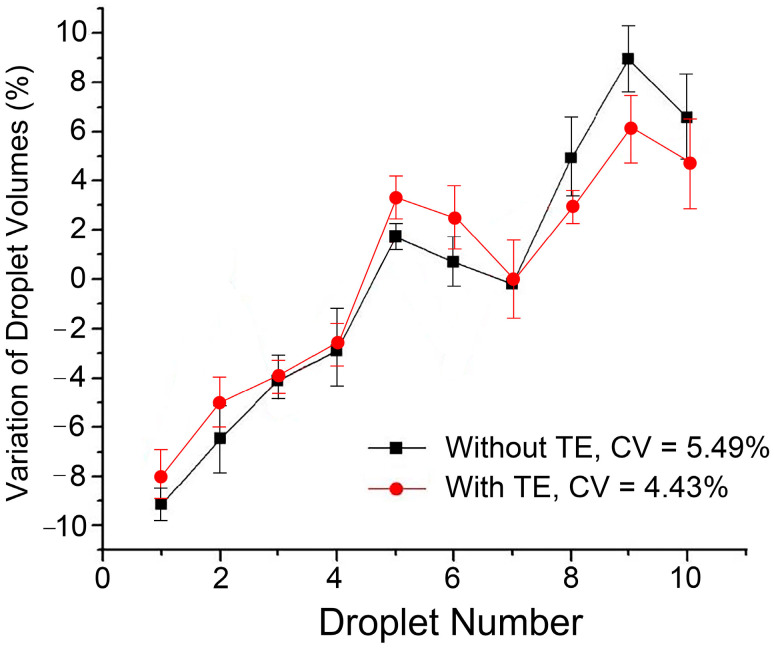
Volumetric variation of consecutively dispensed droplets compared with the average volume for the dispensing process with or without the transitional electrode (TE) for ten repeated experiments.

After one droplet was formed, we used the microscope to collect its images and then drove the droplet along the electrode sequence to the collection pool (electrode 0). For ten droplets dispensed consecutively without re-filling the liquid, the volume variation was measured and demonstrated in Figure 3 while the gap height between the parallel plates was set 160 μm (i.e., two tape layers). The volume of ten droplets produced consecutively is not constant or fluctuating disorderly, but increases monotonically within a certain range. Although several elements like periodic changes in voltage, the contact angle of a droplet on the surface and the morphology of the droplet may impact the process of volume dispensing, further research shows that this monotonically increasing trend is related to the gradual decrease in the volume ratio of the mother droplet in the reservoir to dispensed droplets. 

The force acting on unit per length of contact line was derived by $F=\frac{C}{2}V_{d}^{2}$ with C the capacitance per unit area of the dielectric layer and $V_{d}$ the voltage falling across the dielectric layer [13,14]. As the thickness of the hydrophobic layer on the top plate is negligible compared to the dielectric layer on the bottom plate, $V_{d}$ can be approximated by the voltage applied to actuation electrodes. Assuming *F* remains constant around the contact line on the same electrode, the overall electro-wetting force on the liquid can be calculated by $F_{total}=\int Fds=\frac{C}{2}V_{d}^{2}L_{eff}$ with $L_{eff}$ the projected length of the liquid boundary on the electrode. In the process of droplet generation, the projected area of the water droplets in the reservoir above the reservoir electrode 00 is larger than that above the driving electrode 3, and the electrocapillary force on the liquid is proportional to the projected length of the three-phase line on the actuated electrodes. Therefore, with the continuous generation of droplets, the volume of liquids in the reservoir decreases continuously, and the projected boundary length of the liquid on the reservoir electrode also gradually decreases correspondingly. This tendency leads to a concomitant reduction in the force on the liquid being pulled out during the necking of the drop. On the contrary, since the volume of the drawn liquid is basically unchanged (the projected area is consistent with the sum of the areas of electrode 3 and electrode 4), the projected length of the three-phase line in the corresponding direction is also basically unchanged (roughly equal to the width of the driving electrode 3). So, the electric capillary force that pulls the liquid out is basically constant. Therefore, with the increased order of the dispensed microdroplets, the volume of the mother droplet in the reservoir gradually decreases, the volume of the pulled-out liquid that is sucked back into the reservoir during the necking process gradually decreases, and the volume of dispensed droplets gradually increases with the generation order.

It can be seen from the above analysis that the most direct solution to the problem of droplet volume fluctuation is to continuously replenish the liquid reservoir so that the volume of the liquid in the reservoir remains unchanged. However, the device structure needs to be modified for the continuous replenishment of the liquid reservoir, and the operation is cumbersome. Therefore, it is necessary to improve the structure of the EWOD device itself to improve the volume floating problem of microdroplets.

**Figure 4:**
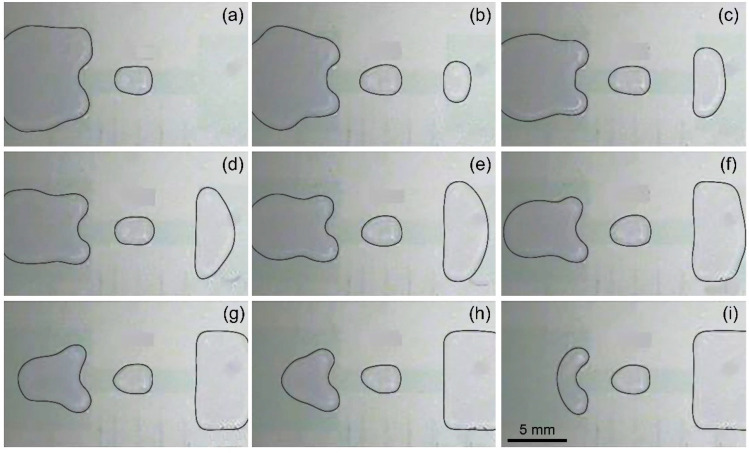
Experimental photos for consecutively dispensed droplets from a non-filling reservoir with a transitional electrode. The droplets are dispensed from left to right with the contour of liquids painted in black. The photos in (**a**–**i**) demonstrate the captured images after the dispensing of the first to the 10th droplets.

Still using the electrode design shown in Figure 1b, electrode 0 and electrode 00 are used as a reservoir electrode and a collection electrode, respectively, and driving electrode 1 is called a transitional electrode (TE). First, a voltage is applied to the driving electrodes 1, 2, and 3 to pull out the liquid from the reservoir. Then, an actuation voltage is applied to the reservoir electrode 0 with the driving electrode 2 connected to the ground, and finally a droplet is formed above the driving electrode 3, as illustrated in Figure 2b. After collecting the image of the droplet, it is driven to the collection pool electrode 00. In this case, the volume fluctuation of 10 microdroplets generated continuously is plotted in Figure 3, and compared with the volume fluctuation of microdroplets without the transition electrode. Moreover, the consecutive dispensing of droplets with the transitional electrode is illustrated by photos in Figure 4.

It can be clearly seen that although the overall trend of the volume of the generated droplets rising with the generation order does not change when the transition electrode is used, the floating range and the coefficient of variation of the droplet volume are both reduced. This improvement occurs as the introduction of transition electrodes reduces the topographical difference at the two ends of the liquid on the splitting electrode (i.e., the driving electrode 2) when the necking process occurs. Through this structure, the impact of the rebound force of the mother droplet and the oscillation effect of the droplet is reduced, which makes it easier to achieve a controllable and consistent droplet contraction shape and confirm the stability of the droplet splitting process. At the same time, the portion of the liquid on the transition electrode reduces the pressure distribution gradient in the pull-out liquid and causes the back-sucking effect of the reservoir to act first on the liquid above the transition electrode. However, due to the short length of the transition electrode, the state of the liquid in the reservoir can still exert an effect on the splitting electrode through the transition electrode, resulting in an increase in the volume of dispensed droplets. 

**Figure 5:**
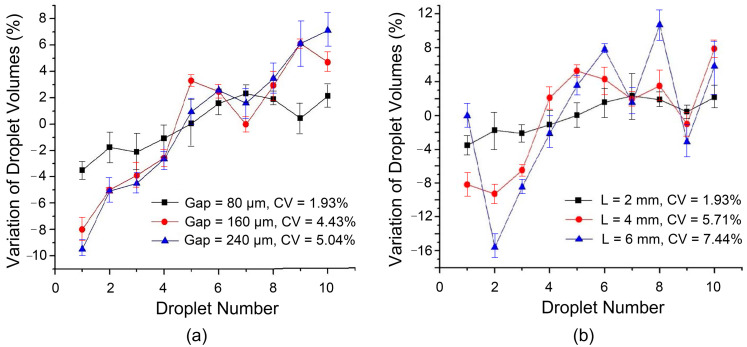
Volumetric variation of consecutively dispensed droplets compared with the average volume for the dispensing process with a transitional electrode for different (**a**) gap heights and (**b**) lengths of the splitting electrode (the width remains 2 mm) in ten repeated experiments.

Based on the computer simulation by Berthier et al. [16], the volumetric consistency of dispensed droplets can be improved by decreasing the gap height between two plates. The smaller gap height results in a weaker influence of various external disturbances on the liquid necking and pinch-off process. Therefore, in this work, three gap heights of 80 μm, 160 μm, and 240 μm were used, corresponding to one, two, and three layers of adhesive tapes between the two plates, respectively. Using the design with transition electrodes and the dispensing process, the volume variation of consecutively dispensed droplets is shown in Figure 5a. It can be clearly seen that the volume of successively generated droplets becomes more stable as the gap height decreases. When the gap height is reduced from 240 μm to 80 μm, the fluctuation of droplet volume decreases from about ±9% to about ±3%, and the coefficient of variation decreases from 5.04% to 1.93%. In fact, reducing the gap width can also reduce the minimum magnitude of the actuation voltage required for droplet generation, which makes the droplet generation process more stable.

Moreover, by optimizing the size (aspect ratio) of the splitting electrode, the instability of the liquid necking processes is reduced and the pinch-off position of the liquid can be more determined. To this end, the droplet generation method with transition electrodes as shown in Figure 2b was adopted, and the width of the parallel bipolar plate gap was set to 80 μm. The dimensions of the other electrodes remain unchanged, and the width of the splitting electrode 2 (on which the liquid necking process occurs) is 2 mm with its length increased from 2 mm, 4 mm, to 6 mm. Volumetric floats are plotted in Figure 5b. 

**Figure 6:**
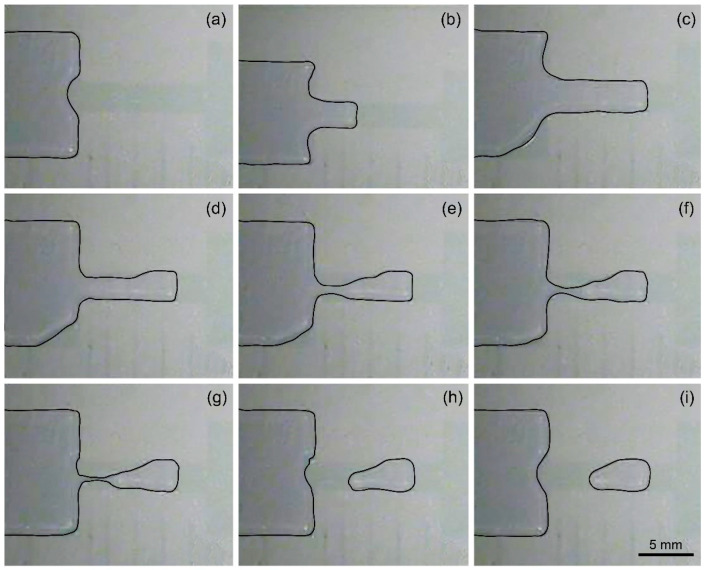
Photos of the necking process with a 6 mm long and 2 mm wide splitting electrode in a time sequence from (**a**) initial state before dispensing to (**i**) a dispensed droplet in its equilibrium shape.

Under the same device parameters and test conditions, the longer the splitting electrode, the greater the uncertainty in the resulting droplet volume. Simply tripling the aspect ratio of the splitting electrode results in a dramatic increase in the coefficient of variation from 1.93% at an aspect ratio of 1 to 7.44% at an aspect ratio of 3. The droplet volume shown in the figure still has an upward trend with the increase in the generation order, but after a few droplets are generated, irregular fluctuations begin to appear. In addition to the gradual reduction in force on the pulled liquid from the gradually decreasing liquid in the reservoir discussed above, the increase in the length of the electrode also increases the uncertainty of the actual splitting position of the liquid. Figure 6 shows the necking and pinching-off processes of the liquid when the splitting electrode obtains an aspect ratio of 3. Since the microstructure and chemical properties of the substrate surface are not completely uniform and the external disturbance to the liquid during the fracture process is not completely symmetrical, the liquid above the splitting electrode does not shrink inward in a symmetrical arc right at the center of the splitting electrode. Conversely, the liquid’s shrinkage pattern and pinch-off position are relatively random. The longer the splitting electrode is, the more uncertain the pinch-off position is, resulting in a larger volume variation of consecutively dispensed droplets. Therefore, the coefficient of variation increases with electrode length, and the accuracy of droplet generation decreases.

As said above, expect the method of decreasing the coefficient of variation. Several other system parameters may impact the process of droplet dispensing. For example, the shape of the splitting electrode may have a great influence on the stability and accuracy of the droplet-splitting process. Round dumbbell-shaped electrodes may advance the stability and accuracy of droplet dispensing since the location of droplet splitting is then under control, though the introduction of extra electrodes with delicate structures complicates the fabrication and operation of devices [23,24]. Moreover, different wettability leads to different volume dispensing processes as well; changing the hydrophilicity of electrode material may benefit decreasing the coefficient of variation. Different hydrophilicity of the electrode surface leads to different contact angles of the droplet on the surface; the hydrophilicity of droplets on the surface determines the effect of the rebound force from the mother droplet; and the contact angle may impact the process of droplet moving and splitting, finally leading to a discrepancy in the droplet volume. Therefore, advancing the material of the electrode can be regarded as another way to decrease the coefficient of variation and improve the volumetric consistency and accuracy of digital microfluidic systems. It is worth mentioning that further study of the original two-plate EWOD structure with square-shaped electrodes saves time and cost in redesigning the electrode shape and eliminates uncertainty about the effect of the new structure on dispensed droplets.

## 4. Conclusions

From the above experiments, it can be found that the volume variation of the droplets consecutively dispensed from a non-filling reservoir can be efficiently alleviated by simply introducing one transition electrode between the reservoir and the liquid splitting position. The effects of the gap height between the two plates and the length of electrodes are also experimentally characterized, which can also optimize the volumetric consistency of droplets without increasing the complexity of the system. This approach introduces no extra external apparatus besides one transitional electrode in the electrode sequence, indicating a simple and reliable approach to improving the volumetric consistency and accuracy of digital microfluidic systems and droplet-based μ-TAS.

## Data Availability

Data is available on request from the authors.
